# Dietary Carbohydrates and ADHD Symptoms: A Systematic Review

**DOI:** 10.3390/nu18101625

**Published:** 2026-05-20

**Authors:** Gabriela Georgieva Panayotova, Antoniya Hachmeriyan

**Affiliations:** Division of Physiology, Department of Physiology and Pathophysiology, Medical University of Varna, 9002 Varna, Bulgaria; antoniyahach@gmail.com

**Keywords:** ADHD, dietary carbohydrates, added sugars, glycemic index, glycemic load, systematic review

## Abstract

Background: Attention-deficit/hyperactivity disorder (ADHD) is a prevalent neurodevelopmental condition with psychosocial impact. Dietary carbohydrates, particularly added sugars, refined starches, and high-glycemic index/load (GI/GL) patterns, have been proposed as modifiable exposures that may relate to attention and behavioral regulation. This systematic review synthesized evidence linking carbohydrate quantity and quality to ADHD-related outcomes. Methods: Following PRISMA 2020, PubMed, Scopus, and Web of Science were searched for English-language studies published from January 2015 to December 2025. Eligible studies included observational and interventional designs in children, adolescents, or adults with a clinical ADHD diagnosis or validated symptom assessment. Risk of bias was assessed using NOS/NOS-adapted criteria, RoB 2, and ROBINS-I. Findings were synthesized narratively by exposure domain. Results: Of 1174 records identified, 48 studies were included: 38 observational and 10 interventional. Sugar-related exposures showed the most consistent pattern: 15 of 16 studies examining added sugars, sugar-sweetened beverages, sweets, candy, or sweet dietary patterns reported positive associations with ADHD diagnosis, symptom severity, hyperactivity, or less favorable ADHD-related outcomes. Findings for total carbohydrate intake were inconsistent. GI/GL-specific evidence was limited but generally adverse in direction. Among intervention studies, symptom improvement after modification was reported in 6 of 10 studies, whereas 4 studies showed mixed, preliminary, non-significant, or non-superior findings. Most observational studies showed moderate to high risk of bias, while interventional studies showed variable risk across domains. Conclusions: Poorer carbohydrate quality may be associated with greater ADHD-related symptom burden, whereas total carbohydrate intake showed inconsistent associations. Certainty remains limited by heterogeneity, residual confounding, risk of bias, and limited carbohydrate-specific intervention evidence.

## 1. Introduction

Attention-deficit/hyperactivity disorder (ADHD) is a common neurodevelopmental condition characterized by persistent patterns of inattention, hyperactivity, and impulsivity that interfere with everyday functioning across educational, social, and occupational domains [1]. Clinical diagnosis is typically established according to standardized DSM-5-TR or ICD-11 criteria, which require persistent, developmentally inappropriate symptoms of inattention and/or hyperactivity–impulsivity for at least 6 months, evidence of several symptoms before 12 years of age, manifestation across more than one setting, clinically significant functional impairment, and exclusion of alternative explanations [2]. Although ADHD is most frequently identified in childhood, it is now well established that the disorder often persists across the life course, with clinically relevant symptoms and impairment extending into adolescence and adulthood [1,3]. Recent evidence syntheses estimate ADHD prevalence at approximately 7.2% in children and adolescents and around 2.5% in adults, although higher estimates have also been reported when broader symptomatic definitions are used [4]. Beyond its core behavioral manifestations, ADHD is associated with functional impairment, reduced quality of life, and unfavorable long-term trajectories involving education, employment, mental health, and physical health [5].

Pharmacological treatment, particularly stimulant medication, remains a cornerstone of ADHD management and has strong evidence for short-term symptom reduction. However, treatment response is heterogeneous, residual symptoms are common, and many patients and families are interested in adjunctive, non-pharmacological strategies that may improve symptom control or broader health outcomes [6,7]. At the same time, ADHD frequently coexists with sleep disturbance, dysregulated eating behaviors, reduced diet quality, and other lifestyle-related factors that may interact with symptom severity and day-to-day functioning [8,9]. These observations have contributed to growing interest in nutritional psychiatry and in the possibility that modifiable dietary exposures may influence ADHD expression, even if they are unlikely to represent a sole causal factor [10,11]. Within this context, dietary carbohydrates represent a particularly relevant exposure domain because they directly influence postprandial glucose regulation and may affect behavioral and neurocognitive processes implicated in ADHD. Importantly, carbohydrate-related exposures are not interchangeable: total carbohydrate intake reflects overall macronutrient quantity, whereas added/free sugars, refined starches, low-fiber carbohydrate sources, and high- glycemic index/glycemic load (GI/GL) foods capture dimensions of poorer carbohydrate quality.

ADHD has most often been examined in relation to overall dietary patterns, micronutrient status, supplementation, elimination diets, and more recently the gut microbiota [10,11,12]. Broadly, “healthy” dietary patterns rich in fruits, vegetables, and minimally processed foods have tended to show inverse associations with ADHD, whereas “Western diet” or “junk food” patterns characterized by confectionery, soft drinks, refined grains, and highly processed foods have more often been associated with greater symptom burden [11,13]. However, prior reviews also emphasize that the direction of this relationship remains uncertain. Children or adults with more severe ADHD symptoms may be more likely to adopt poorer dietary habits because of emotional eating, irregular meal timing, reward-driven food choices, family stress, or medication-related appetite effects. As a result, the diet–ADHD association is plausibly bidirectional, and the contribution of specific dietary components remains insufficiently resolved [10,11,14].

This carbohydrate-focused perspective is physiologically justified because carbohydrates are the principal dietary determinants of postprandial glucose dynamics, and their effects depend not only on quantity but also on quality, including the degree of processing, fiber content, and GI/GL [15,16]. This distinction may be especially relevant in ADHD because the neural systems underlying attention, behavioral inhibition, reward processing, and executive control are metabolically demanding and potentially sensitive to dysregulated glucose availability [17]. More broadly, alterations in glucose homeostasis have been linked to adverse effects on cognition and brain development, while evidence from cognitive nutrition research suggests that lower-glycemic-load meals may support aspects of memory and attention under some conditions, particularly later in the postprandial period [18,19]. Although these data are not ADHD-specific, they provide a plausible mechanistic rationale for examining whether carbohydrate quality may influence attentional performance and behavioral regulation in susceptible individuals.

A carbohydrate-centered perspective is also warranted because not all carbohydrate exposures are biologically equivalent. Total carbohydrate intake may be too crude a metric to capture clinically meaningful differences, as diets with similar total carbohydrate content can differ substantially in added sugars, refined starches, dietary fiber, whole-grain content, and glycemic properties [20,21,22]. Added sugars and sugar-sweetened beverages may promote rapid glycemic excursions, high palatability, and overconsumption, whereas higher-fiber and whole-grain carbohydrate sources are generally associated with slower absorption, greater satiety, and more favorable metabolic and inflammatory profiles [23,24]. In support of this distinction, a previous meta-analysis found a positive overall association between sugar and sugar-sweetened beverage consumption and ADHD symptoms, while underscoring the need to distinguish carbohydrate quantity from carbohydrate quality more clearly [25].

Several mechanisms may link carbohydrate quality with ADHD symptom expression. Highly refined or high-GI/GL carbohydrate patterns can promote rapid glucose excursions and greater glycemic variability, with potential downstream effects on alertness, fatigue, irritability, and cognitive efficiency [18,26]. Highly palatable sugar-rich foods may also interact with impulsivity, reward sensitivity, and emotional eating, while carbohydrate quality may indirectly influence sleep and appetite regulation, both of which are clinically relevant in ADHD [10,11,27,28]. Conversely, fiber-rich and less-refined carbohydrate sources may support gut microbial support and inflammatory signaling, providing a plausible gut–brain pathway [12,29,30,31].

Despite these plausible mechanisms, the evidence base remains fragmented. Existing studies differ substantially in population characteristics, diagnostic definitions, age groups, dietary assessment methods, exposure categorization, confounder adjustment, and outcome measurement. Some studies focus on total sugars or sugar-sweetened beverages, others on dietary patterns, and others still on GI/GL, fiber, or meal composition. Interventional studies are comparatively fewer and often constrained by short duration, small sample size, inconsistent adherence reporting, and limited standardization of dietary protocols [10,11]. In addition, much of the literature has been interpreted through the broader lens of ‘healthy’ versus ‘Western’ dietary patterns, which may obscure the independent contribution of carbohydrate-related exposures [32]. Recent reviews on ADHD and nutrition have therefore called for more precise, exposure-specific syntheses and for more rigorous studies capable of disentangling causality from confounding and reverse causation [11].

Against this background, a focused systematic review of dietary carbohydrates and ADHD symptoms is justified. Rather than treating carbohydrates as a single nutritional category, it is important to distinguish total carbohydrate intake from specific indicators of carbohydrate quality, such as added sugars, refined carbohydrates, fiber intake, whole-grain consumption, and dietary GI/GL. Such an approach may help clarify whether the observed associations in the literature are driven primarily by excess carbohydrate exposure overall, by poorer carbohydrate quality, or by broader unhealthy eating patterns in which carbohydrate-rich ultra-processed foods are prominent. It may also help identify where findings are most consistent, where evidence is weakest, and which subdomains are most promising for future intervention research. Clarifying these relationships has practical relevance because dietary guidance targeting carbohydrate quality may represent a feasible adjunct to standard ADHD management, particularly when framed as supportive nutritional care rather than as a replacement for evidence-based pharmacological or behavioral treatment. Current ADHD clinical guidelines generally emphasize balanced diet, good nutrition, and broader lifestyle support, but provide limited carbohydrate-specific recommendations regarding total carbohydrate intake, added sugars, refined carbohydrates, fiber, or glycemic quality [33].

Importantly, this review does not assume that high carbohydrate intake causes ADHD. Rather, it examines whether different carbohydrate-related exposures are associated with ADHD diagnosis, symptom severity, related behavioral outcomes, or symptom change following dietary intervention. Therefore, the aim of the present systematic review is to synthesize the available evidence on the relationship between dietary carbohydrate quantity and quality and ADHD symptoms across observational and interventional studies. Specifically, it evaluates associations involving total carbohydrates, sugars and added sugars, starch, dietary fiber, glycemic index, glycemic load, and refined versus whole-grain carbohydrate patterns, and considers the extent to which these exposures are linked to ADHD symptom severity or related behavioral outcomes. By consolidating this evidence, the review seeks to clarify current knowledge, highlight methodological limitations, and define priorities for future nutritional research in ADHD.

## 2. Materials and Methods

### 2.1. Study Design and Reporting Framework

This systematic review was undertaken to synthesize the available evidence on the relationship between dietary carbohydrate quantity and quality and ADHD symptoms across the lifespan. The review was designed and reported in accordance with the Preferred Reporting Items for Systematic Reviews and Meta-Analyses (PRISMA) 2020 statement. The review question, eligibility criteria, search strategy, screening process, data extraction plan, and synthesis approach were specified in advance to enhance methodological transparency and reproducibility. The protocol was prospectively registered in the International Prospective Register of Systematic Reviews (PROSPERO; CRD420261359471). No substantive deviations from the registered protocol occurred; methodological refinements made during the review process are reported transparently in the relevant Methods subsections.

### 2.2. Eligibility Criteria

Eligibility criteria were defined according to the Population–Exposure–Comparator–Outcome–Study design (PECOS) framework.

Population. Studies were eligible if they included children, adolescents, or adults of any sex with either: (1) a clinical diagnosis of ADHD based on recognized diagnostic criteria, including the Diagnostic and Statistical Manual of Mental Disorders (DSM) or the International Classification of Diseases (ICD); or (2) ADHD symptoms assessed using validated rating scales or standardized instruments. Community, school-based, outpatient, and clinical populations were eligible. Studies including comparison groups without ADHD were also eligible, provided that ADHD-related outcomes were reported separately or could be clearly identified.

Exposure/intervention. Eligible studies examined one or more carbohydrate-related dietary exposures or interventions, including total carbohydrate intake, total sugars, added or free sugars, sugar-sweetened beverages, starch, dietary fiber, glycemic index (GI), glycemic load (GL), refined carbohydrate intake, whole-grain intake, or dietary patterns in which carbohydrate quality was a clearly identifiable component. Both observational exposures and dietary interventions targeting carbohydrate quality or carbohydrate-related dietary modification were eligible.

Comparator. Comparators were defined according to study design. In observational studies, eligible comparators included lower versus higher carbohydrate-related exposure groups, non-ADHD control groups, or groups differing in ADHD symptom severity where carbohydrate-related exposures were compared. In interventional studies, eligible comparators included usual diet, control diet, placebo, no intervention, or alternative dietary interventions, where applicable.

Outcomes. The primary outcome was ADHD symptom severity, including total symptom burden and domain-specific symptoms of inattention and hyperactivity/impulsivity, as measured using validated ADHD diagnostic criteria or standardized rating instruments. Secondary outcomes included related behavioral and neuropsychological outcomes reported in direct connection with ADHD symptomatology, including executive function, attentional performance, impulsivity, and emotional/behavioral dysregulation. Additional outcomes, where reported, included categorical ADHD diagnosis/likelihood, changes in ADHD symptom subscale scores over time, and neurocognitive outcomes related to attention, inhibitory control, or behavioral regulation when these were reported separately from primary ADHD symptom scales.

Study design. Eligible study designs included randomized controlled trials, non-randomized interventional studies, cohort studies, case–control studies, and cross-sectional observational studies. Narrative reviews, systematic reviews, meta-analyses, editorials, letters, expert opinions, conference abstracts without full data, case reports, case series, animal studies, and in vitro studies were excluded.

Other restrictions. Only English-language studies published between 1 January 2015 and 31 December 2025 were eligible.

### 2.3. Information Sources and Search Strategy

Electronic searches were conducted in PubMed, Scopus, and Web of Science Core Collection. The final searches were conducted on 5 April 2026 and covered studies published from 1 January 2015 to 31 December 2025. Searches were restricted to English-language publications. The 2015 lower date limit was selected to capture a contemporary evidence window of approximately the most recent decade of research, reflecting current dietary patterns, food environments, ADHD diagnostic practices, and methodological standards in nutritional epidemiology. The English-language restriction was applied for feasibility and consistency of full-text assessment, although it is acknowledged as a potential source of language bias. No age-group filters were applied, because eligible populations included children, adolescents, and adults. No study-design filters were applied at the search stage; study-design eligibility was assessed during title/abstract and full-text screening. The review was restricted to human studies. Animal and in vitro studies were excluded during title/abstract and full-text screening according to the predefined eligibility criteria.

Search strategies were developed for each database using two concept blocks combined with the Boolean operator AND: ADHD-related terms and carbohydrate-related dietary exposure terms. The ADHD block included “attention-deficit/hyperactivity disorder”, ADHD, “attention deficit hyperactivity disorder”, “attention deficit”, and hyperactivity. The carbohydrate-related block included carbohydrate*, “dietary carbohydrate*”, sugar*, “added sugar*”, “free sugar*”, sucrose, fructose, starch, fiber/fibre, “dietary fiber/fibre”, “glycemic/glycaemic index”, “glycemic/glycaemic load”, “refined grain*”, “whole grain*”, and “sugar-sweetened/sugar sweetened beverage*”.

The PubMed search used Title/Abstract fields and publication-date and English-language restrictions; Scopus used TITLE-ABS-KEY fields with publication-year and English-language restrictions; and Web of Science used Topic fields with publication-year and English-language restrictions. Database-specific syntax was adapted to the indexing and field conventions of each database. In addition to database searching, reference lists of included studies and relevant reviews were screened manually to identify further eligible studies.

### 2.4. Study Selection

All retrieved records were imported into reference-management software, and duplicate records were removed prior to screening. Screening was conducted in two stages: (1) title and abstract screening; and (2) full-text assessment of potentially eligible articles. Study selection was conducted independently by two reviewers, and disagreements were resolved through discussion until consensus was reached. Inter-reviewer agreement was not quantified using Cohen’s kappa because independent screening-decision logs were not retained in a format allowing reliable post hoc calculation. This was considered when reporting the screening process as a methodological limitation. Reasons for exclusion at the full-text stage were recorded. The study selection process was documented using a PRISMA 2020 flow diagram.

### 2.5. Data Extraction

Data were extracted independently by two reviewers using a predefined standardized extraction form. Before full data extraction, the extraction form was pilot-tested and calibrated on an initial subset of included studies [10%]. Both reviewers independently extracted data from this subset, compared entries, resolved discrepancies through discussion, and refined the extraction form and coding rules before proceeding with full extraction. The following information was collected from each included study: first author and year of publication; country and study setting; study design; sample size; age group and participant characteristics; ADHD diagnostic method or symptom assessment tool; dietary assessment method; carbohydrate-related exposure(s) or intervention(s); comparator or control group, where applicable; outcome measures; covariates or confounders included in adjusted analyses; principal findings, including reported effect estimates; and main study limitations noted by the authors or identified during review. For intervention studies, additional information was extracted on the dietary protocol, duration of intervention, adherence assessment, and outcome timing. No additional information was sought from study investigators beyond the published reports.

Potentially overlapping populations were identified by comparing cohort name, country, recruitment period, study setting, sample characteristics, author group, and sample size. Multiple reports from the same or overlapping datasets were retained only when they addressed distinct exposures, outcomes, developmental periods, or analytic questions; otherwise, the most comprehensive or most recent report was prioritized. Because no quantitative meta-analysis was performed, overlapping datasets were considered qualitatively to avoid over-weighting evidence from the same underlying population.

### 2.6. Risk of Bias and Study Quality Assessment

Risk-of-bias judgments were categorized according to each tool: RoB 2 as low risk, some concerns, or high risk of bias; ROBINS-I as low, moderate, serious, critical risk of bias, or no information; and NOS/NOS-adapted assessments as low, moderate, or high risk of bias. The appraisal results were summarized in structured methodological-quality and risk-of-bias figures and used to guide the narrative synthesis. Greater interpretive weight was given to studies with clearer exposure definitions, stronger confounder adjustment, prospective or interventional designs, and lower risk of bias, whereas findings from cross-sectional, poorly adjusted, or higher-risk studies were interpreted cautiously.

Publication bias and small-study effects were not formally assessed using funnel plots or statistical tests because no quantitative meta-analysis was performed and the included studies were highly heterogeneous in design, exposures, ADHD outcomes, and effect metrics. Instead, potential publication bias was considered narratively, particularly when positive findings came from small or methodologically limited studies.

A formal outcome-level GRADE assessment was not performed because of substantial methodological and clinical heterogeneity and the absence of quantitative pooling. However, certainty of evidence was considered narratively using GRADE-informed domains, including risk of bias, inconsistency, indirectness, imprecision, and potential publication or language bias.

### 2.7. Outcomes and Effect Measures

Outcome data were extracted for any reported assessment time point, including cross-sectional assessments and baseline/follow-up measurements in longitudinal or interventional studies. For synthesis, priority was given to validated ADHD-specific scales and symptom subscales reflecting inattention and hyperactivity/impulsivity. Where studies reported multiple behavioral outcomes, ADHD-specific outcomes were prioritized over broader behavioral constructs unless those constructs were explicitly embedded within validated ADHD assessment frameworks.

### 2.8. Data Synthesis

Because substantial heterogeneity was anticipated in study populations, age groups, exposure definitions, dietary assessment methods, ADHD outcome measures, and study design, a structured narrative synthesis was planned a priori rather than a quantitative meta-analysis. Studies were grouped according to the main carbohydrate-related exposure domain: (1) total carbohydrate intake; (2) sugars and added sugars; (3) glycemic index and glycemic load; (4) fiber and whole-grain intake; and (5) interventions targeting carbohydrate quality or carbohydrate-related dietary modification. Findings were additionally considered according to age group, study design, and direction of association (positive, inverse, or null/inconsistent). For observational evidence, a positive association indicated that higher exposure was associated with greater ADHD symptom severity, higher odds of ADHD diagnosis, or less favorable ADHD-related outcomes. Аn inverse association indicated lower symptom burden or more favorable ADHD-related outcomes with higher exposure, whereas mixed/null findings indicated non-significant, inconsistent, or directionally divergent results across models, outcomes, or subgroups.

Interpretation emphasized consistency of direction, exposure specificity, adequacy of confounder adjustment, biological plausibility, and overall methodological robustness. Greater interpretive weight was given to studies with clearer exposure definition, stronger confounder adjustment, lower risk of bias, and more rigorous study design. Studies with minimal or no adjustment for key confounders, such as age, sex, socioeconomic status, medication use, physical activity, sleep, body mass index, total energy intake, or overall diet quality, were not excluded from the synthesis, but were interpreted with lower confidence and given less interpretive weight because of the increased risk of residual confounding.

Cross-sectional evidence was interpreted cautiously because temporality could not be established, and broader dietary-pattern studies were interpreted with caution unless carbohydrate-related exposures could be clearly isolated.

Because a quantitative meta-analysis was not performed, formal statistical sensitivity analyses were not applicable. Instead, risk-of-bias judgments were used as an interpretive sensitivity framework when weighing the consistency and robustness of findings across exposure domains.

## 3. Results

### 3.1. Study Selection

The database search identified a total of 1174 records across PubMed, Scopus, and Web of Science. After removal of duplicates, the remaining records were screened by title and abstract for relevance to the review question. Full texts of potentially eligible articles were then assessed against the predefined inclusion and exclusion criteria. Following this process, 48 studies were included in the final qualitative synthesis, comprising 38 observational studies and 10 interventional studies.

**Figure 1:**
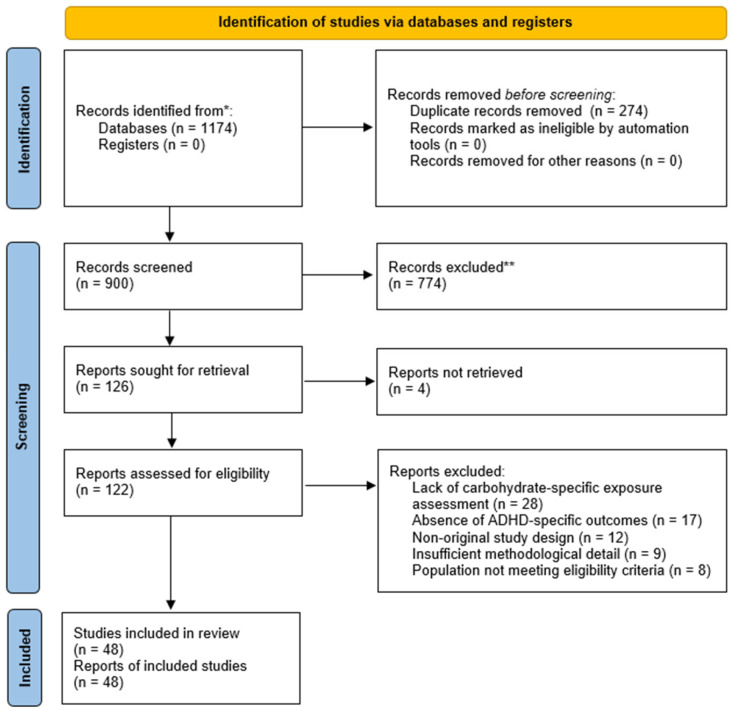
PRISMA flowchart showing studies identification, screening, and inclusion process. * Records were identified through database searching in PubMed, Scopus, and Web of Science; no registers were searched. ** Records were excluded during title and abstract screening because they did not meet the predefined eligibility criteria.

The main reasons for exclusion at the full-text stage included lack of carbohydrate-specific exposure assessment, absence of ADHD-specific outcomes, non-original study design, insufficient methodological detail, and failure to meet the predefined population criteria. A detailed overview of the study identification and selection process is presented in the PRISMA 2020 flow diagram (Figure 1).

### 3.2. Study Characteristics

The 48 included studies showed substantial diversity in design, population characteristics, exposure definition, and outcome assessment. Most studies were observational in nature, including cross-sectional, case–control, and prospective cohort designs, while a smaller subset consisted of dietary intervention trials evaluating the effects of carbohydrate-related dietary modification on ADHD symptoms or related behavioral outcomes.

The included literature covered children and adolescents predominantly, with fewer studies involving adult participants. ADHD was identified either through formal clinical diagnosis based on recognized diagnostic criteria or through validated symptom scales assessing inattentive, hyperactive–impulsive, or combined symptom dimensions. Outcome measures varied across studies and included parent-reported, teacher-reported, clinician-rated, and self-reported instruments, as well as, in some cases, neurobehavioral or executive function measures related to ADHD symptomatology.

Dietary assessment methods also varied considerably. These included food frequency questionnaires, 24 h dietary recalls, dietary records, structured dietary interviews, and broader dietary pattern instruments. Carbohydrate-related exposures were operationalized heterogeneously and included:

- total carbohydrate intake,
- total or added sugar intake,
- consumption of sugar-sweetened beverages,
- dietary glycemic index and glycemic load,
- starch intake,
- fiber intake,
- refined carbohydrate consumption,
- whole-grain intake, and
- dietary patterns in which carbohydrate quality constituted a meaningful component.

This heterogeneity in both exposure and outcome assessment limited direct comparability across studies and complicated quantitative pooling. A summary of the characteristics of the included observational and interventional studies is provided in Table 1 and Table 2, respectively.

Across the 38 observational studies, the direction of findings varied by exposure domain. Sugar-related exposures showed the most consistent pattern: 15 of 16 studies examining added sugars, sugar-sweetened beverages, sweets, candy, or sweet dietary patterns reported positive associations with ADHD diagnosis, symptom severity, hyperactivity, or less favorable ADHD-related behavioral outcomes. In contrast, studies examining total carbohydrate intake or broad carbohydrate intake showed mixed results, with several reporting null or inconsistent associations. GI/GL-specific evidence was less extensive, but the available studies generally reported adverse associations between higher glycemic exposure and ADHD-related outcomes. Studies examining fiber-rich, whole-grain, Mediterranean-style, plant-based, or MIND-style dietary patterns more often reported inverse or favorable associations, although these exposures usually reflected broader diet quality rather than carbohydrate quality alone. Among the 10 intervention studies, 6 reported ADHD symptom improvement after dietary modification, while 4 showed mixed, preliminary, non-significant, or non-superior findings. These descriptive proportions were used to improve transparency of the narrative synthesis and should not be interpreted as pooled effect estimates.

### 3.3. Methodological Quality and Risk of Bias

Methodological quality varied across the included studies and should be considered when interpreting the overall evidence base. Overall, most observational studies were rated as having moderate to high risk of bias, whereas randomized controlled trials and non-randomized interventional studies showed variable risk across domains, particularly regarding randomization, deviations from intended interventions, confounding, adherence, and outcome measurement.

Risk-of-bias appraisal was conducted using design-specific tools, with RoB 2 applied to randomized controlled trials, ROBINS-I to non-randomized interventional studies, and the Newcastle–Ottawa Scale (NOS) or an NOS-adapted structured summary to observational studies, as appropriate. Overall, the available evidence was characterized by substantial methodological heterogeneity, particularly with respect to study design, exposure definition, dietary assessment, outcome ascertainment, and confounder control.

**Figure 2:**
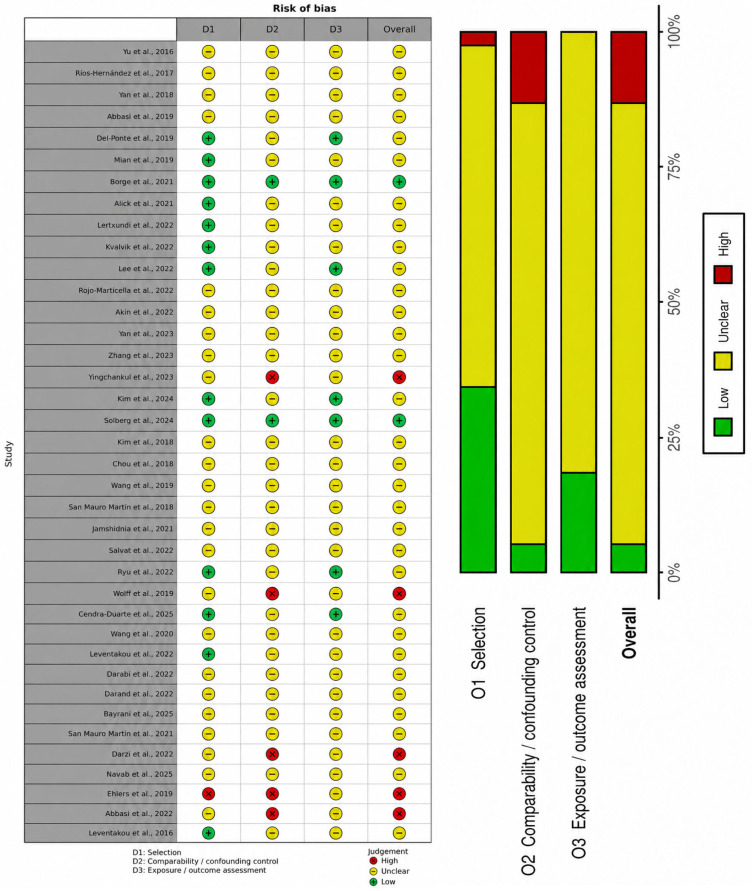
Methodological-quality summary of observational studies assessed using NOS-based domains [14,34,35,36,37,38,39,40,41,42,43,44,45,46,47,48,49,50,51,52,53,54,55,56,57,58,59,60,61,62,63,64,65,66,67,68,69,70].

Among the observational studies, the evidence base was dominated by cross-sectional designs, which limited causal inference and made it difficult to establish the temporal direction of the association between carbohydrate-related dietary exposures and ADHD symptoms. In many studies, dietary intake and symptom burden were assessed at the same time point, increasing the possibility of reverse causation, whereby more severe ADHD symptoms may influence dietary choices rather than result from them. Additional concerns included reliance on self-reported or parent-reported dietary intake, variation in dietary instruments, inconsistent distinction between total sugars and added sugars, and incomplete adjustment for relevant confounders such as socioeconomic status, sleep, medication use, body mass index, physical activity, and overall diet quality. ADHD outcomes were also not uniformly defined, with some studies relying on formal clinical diagnosis and others on symptom questionnaires or screening instruments. The methodological quality summary for observational studies is presented in Figure 2.

**Figure 3:**
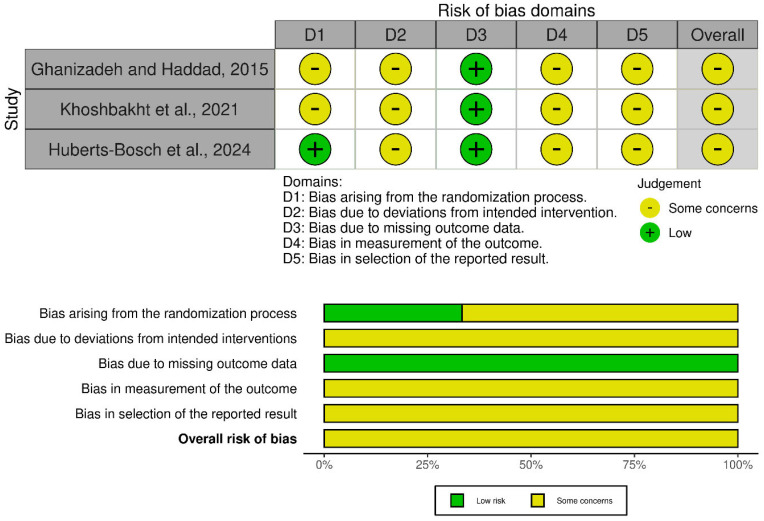
Risk-of-bias assessment of randomized controlled trials using RoB 2 [71,76,79].

The interventional studies were also heterogeneous in quality. The randomized trials generally showed clearer intervention definitions but still varied in randomization reporting, outcome measurement, and reporting transparency. The corresponding RoB 2 assessment is shown in Figure 3.

**Figure 4:**
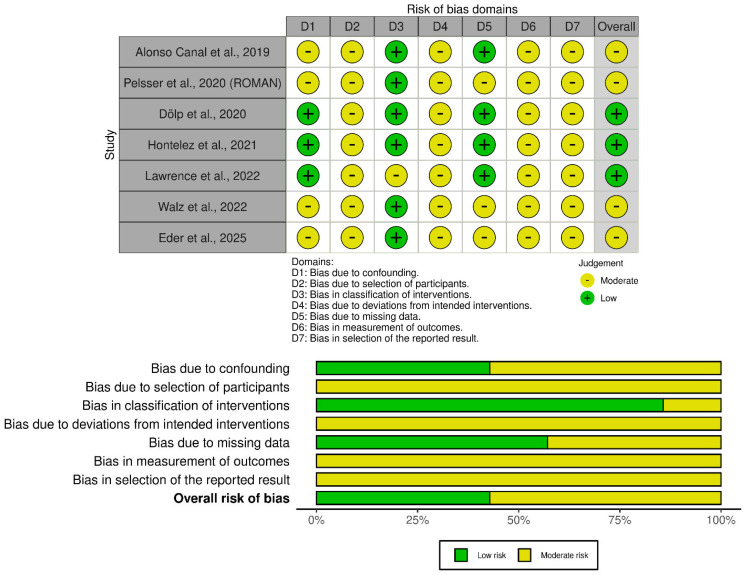
Risk-of-bias assessment of non-randomized interventional studies using ROBINS-I [72,73,74,75,77,78,80].

Non-randomized interventional studies were more vulnerable to bias arising from confounding, participant selection, adherence-related deviations, and outcome measurement, particularly in open-label, pilot, or follow-up designs. These judgments are summarized in Figure 4.

Across intervention studies overall, common limitations included relatively small sample sizes, short intervention duration, and variability in the degree to which carbohydrate quality was specifically modified. In several cases, carbohydrate-related changes occurred within broader dietary interventions, making it difficult to isolate the specific contribution of carbohydrate exposure to observed symptom changes [71,73,74,75,76,77,79,80].

Taken together, the quality appraisal indicates that the available evidence should be interpreted cautiously, particularly where positive associations arose from studies with limited exposure specificity, residual confounding, or insufficient follow-up. These methodological limitations do not negate the observed pattern of findings, but they do reduce certainty and reinforce the need for larger, better-controlled prospective and interventional studies.

### 3.4. Total Carbohydrate Intake

Findings regarding total carbohydrate intake were generally inconsistent across the included studies. Total carbohydrate consumption did not emerge as a uniformly reliable correlate of ADHD diagnosis or symptom severity. Some studies suggested that higher carbohydrate intake or carbohydrate-rich unhealthy eating patterns were associated with greater symptom burden, whereas others found no clear association after adjustment for potential confounders [14,36,37,38,43,46,53]. In a smaller number of studies, mixed associations were observed depending on population, study design, or the dietary instrument used [52,60,61,65,70]. Overall, the available evidence did not support a consistent association between total carbohydrate intake and ADHD symptoms.

### 3.5. Sugars, Added Sugars, and Sugar-Sweetened Beverages

A more consistent pattern emerged for sugars, added sugars, and sugar-sweetened beverages. Across observational studies, higher intake of sugar-sweetened beverages, sweets, candy, and sweet dietary patterns was more frequently associated with ADHD diagnosis, hyperactivity, or greater symptom severity. This pattern was observed in both cross-sectional and longitudinal studies and was evident in several pediatric and adolescent populations, with one study in young adults also reporting a positive association between added sugar from beverages and ADHD symptoms [34,35,43,47,48,49,66]. However, not all sugar-related findings were uniform, and some broader dietary-pattern studies made it difficult to isolate the independent contribution of sugar exposure from other co-occurring dietary characteristics [14,35,36,46,53,56].

### 3.6. Glycemic Index and Glycemic Load

Evidence specifically addressing dietary glycemic index and glycemic load was less extensive but directionally suggestive. The available cohort findings indicated that higher maternal or early-life glycemic loading was associated with less favorable offspring attention- or ADHD-related behavioral outcomes [40,41,42,59]. In addition, studies of sweet or highly processed dietary patterns were broadly consistent with the possibility that poorer glycemic quality may be relevant to ADHD symptom expression. Although the GI/GL evidence base remains smaller than that for added sugars or sugar-sweetened beverages, the direction of association was more often adverse than protective [36,40,43,59].

### 3.7. Fiber, Whole Grains, and Higher-Quality Carbohydrate Patterns

Evidence on fiber, whole grains, and higher-quality carbohydrate patterns was comparatively limited but tended to suggest an inverse association with ADHD-related outcomes. Lower prenatal fiber intake was associated with higher offspring ADHD symptom levels, while healthier dietary patterns such as Mediterranean, plant-based, or MIND-style patterns were more often linked to lower ADHD odds or lower symptom burden [35,50,62,63,64,65,67]. Nevertheless, these patterns reflect broader overall diet quality rather than carbohydrate quality alone, and therefore should be interpreted cautiously when considering the independent role of carbohydrate-related exposures.

### 3.8. Interventional Evidence

**Figure 5:**
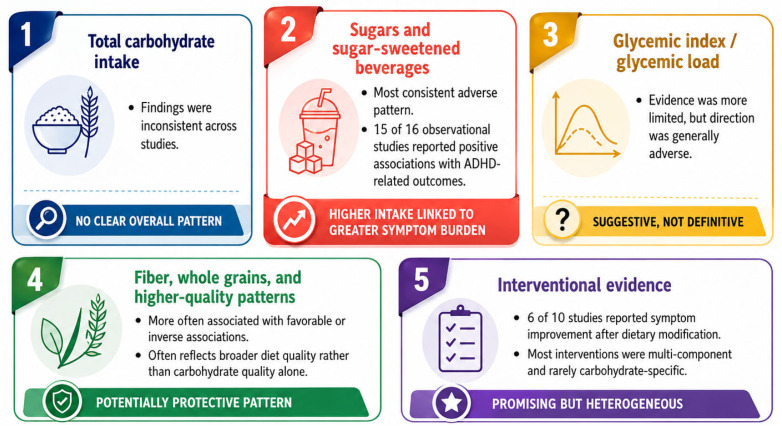
Summary of carbohydrate-related evidence in ADHD.

While observational evidence suggested relatively consistent adverse associations for sugar-related exposures, interventional findings were less consistent and rarely carbohydrate-specific. Several studies of few-foods diets, oligoantigenic diets, and broader structured dietary interventions reported symptomatic improvement, including reductions in ADHD symptom severity or clinically meaningful responder rates [73,74,75,76,78,80]. A DASH-style diet also showed promising findings in one randomized trial [76]. However, not all interventions showed clear superiority, and in one recent trial elimination and healthy-diet approaches both showed responses without a clear advantage of the elimination strategy [79]. Importantly, most intervention studies altered multiple aspects of diet simultaneously, which limits attribution of observed effects specifically to carbohydrate quantity or carbohydrate quality [71,72,73,74,76,77,79]. Short follow-up, small sample sizes, and inconsistent adherence reporting further reduced interpretability. A visual summary of the main exposure-specific findings is presented in Figure 5.

## 4. Discussion

The present systematic review indicates that carbohydrate quality, rather than total carbohydrate quantity, is more consistently associated with ADHD-related outcomes. The most consistent adverse signal was observed for added sugars, sugar-sweetened beverages, sweet dietary patterns, and poorer glycemic characteristics, whereas findings for total carbohydrate intake were inconsistent. This suggests that carbohydrates should not be interpreted as a uniform exposure in ADHD research, but in relation to food source, processing level, fiber content, and glycemic properties. However, it remains unclear whether carbohydrate quality has independent effects or primarily reflects broader dietary patterns and overall diet quality.

Importantly, the findings do not support the conclusion that a high-carbohydrate diet causes ADHD. Rather, poorer carbohydrate quality may be associated with greater ADHD-related symptom burden or less favorable behavioral regulation in some populations. Because studies varied in outcome measurement and often reported ADHD diagnosis/likelihood, total symptom scores, hyperactivity, or broader behavioral outcomes, the evidence does not allow firm conclusions about effects on inattention versus hyperactivity/impulsivity.

This pattern was particularly evident for sugar-rich exposures. Several observational studies reported positive associations between sugar-sweetened beverages, sweets, candy, or sweet dietary patterns and ADHD diagnosis or symptom severity [34,35,36,43,47,48,49,53]. Although effect sizes and exposure definitions varied, the overall direction was relatively coherent. These findings are consistent with the earlier meta-analysis by Farsad-Naeimi et al., which reported a positive pooled association between overall sugar/sugar-sweetened beverage consumption and ADHD symptoms, albeit with substantial heterogeneity, and with the more recent meta-analysis by Khazdouz et al., which likewise found that junk-food consumption—especially sweetened beverages/soft drinks and sweets/candies—was positively associated with ADHD symptoms [25,81].

At the same time, the present review suggests that the literature becomes less consistent when carbohydrate exposure is assessed only as total carbohydrate intake. This is an important distinction. Total carbohydrate intake may be too crude an exposure metric to capture biologically meaningful variation, because diets with similar total carbohydrate content can differ substantially in added sugar content, fiber density, food processing level, and glycemic characteristics. This likely explains why some studies showed positive associations for sugar-rich or refined patterns, whereas Del-Ponte et al. (2019), for example, did not observe a clear association between sucrose intake and incident ADHD [14,35,36,43,51,53,55]. Taken together, the available evidence argues against interpreting “carbohydrates” as a single etiologic category in ADHD research.

The smaller but increasingly relevant body of evidence on glycemic quality also deserves attention. In the included studies, higher maternal glycemic loading or higher maternal dietary glycemic index/glycemic load was associated with less favorable offspring attention- or ADHD-related behavioral outcomes. Although the number of GI/GL-specific studies remains limited, their direction of association is broadly compatible with the hypothesis that poorer glycemic quality may be relevant to attentional and behavioral regulation [40,41,42,59]. These findings are physiologically compatible with the introductory rationale linking high-GI/GL exposure to glycemic variability and neurobehavioral regulation. However, the evidence base remains too small to support firm conclusions regarding GI/GL as an independent risk factor.

Additional biological pathways may also be relevant. ADHD is closely linked to impairments in attention, inhibitory control, impulsivity, and reward-related behavior, and highly palatable sugar-rich foods may interact with reward sensitivity, emotional eating, and reinforcement-driven food choice [1,3,9,10,11,27]. Glucose metabolism and glycemic regulation are also relevant to brain development and cognitive function, suggesting that repeated high-glycemic exposure and glycemic fluctuations could plausibly influence attention and executive regulation in susceptible individuals [17,18,19,26]. Finally, many sugar- and refined-carbohydrate exposures occur within broader ultra-processed or “junk-food” dietary patterns. Therefore, observed associations may partly reflect high energy density, low fiber content, high palatability, additives, and displacement of nutrient-dense foods rather than carbohydrate exposure alone [13,14,25,81,82,83,84,85].

Evidence regarding fiber, whole grains, and healthier carbohydrate-related dietary patterns was more suggestive than definitive. Solberg et al. (2024) reported that lower prenatal fiber intake was associated with higher offspring ADHD symptom levels, while inverse associations were observed for healthier dietary patterns in studies such as San Mauro Martín et al. (2021), Darabi et al. (2022), Darand et al. (2022), and Bayranj et al. (2025) [50,62,63,64,65]. Better maternal or child diet quality was also linked with slightly more favorable outcomes [39]. Nevertheless, these findings should be interpreted cautiously, because these exposures usually reflect broader overall diet quality rather than isolated carbohydrate quality. In other words, the apparent protective signal may partly reflect healthier dietary matrices, lower ultra-processed food intake, or correlated family and lifestyle factors rather than carbohydrate quality alone.

The intervention literature added а nuance but did not establish a clearly carbohydrate-specific treatment effect. Positive symptom changes were reported in several studies of few-foods or oligoantigenic dietary approaches [73,74,75,78,80], and one randomized trial suggested benefits from a DASH-style diet [76]. However, these interventions modified multiple components of diet simultaneously, often including elimination of processed foods, additives, common triggers, or broader dietary restructuring. Accordingly, they provide support for the clinical relevance of diet in some subgroups of ADHD, but they do not isolate the independent effect of carbohydrate quantity or glycemic quality. This interpretation is consistent with broader recent reviews, which have suggested that few-foods diets may be promising in selected children with ADHD, while emphasizing the need for larger and better-controlled studies [10,11,82,86,87].

Another important issue is the likely bidirectionality of the diet–ADHD relationship. Several studies in the present review suggest that poorer dietary quality may accompany greater ADHD symptom burden, but at least one included cohort study found that ADHD symptoms predicted lower later diet quality rather than the reverse [35,36,37,38,39,51,53,56,62,65]. This raises the possibility of reverse causation, particularly in cross-sectional studies. Children or adolescents with greater impulsivity, irregular eating patterns, emotional dysregulation, or family-level stress may be more likely to consume highly palatable, sugary, and ultra-processed foods [38,44,45,58,61,68,70]. Consequently, observed associations may reflect not only a potential dietary contribution to symptom burden, but also symptom-driven food choice [38,45,58,61,68,70]. Residual confounding may also arise from ADHD medication use, particularly appetite-suppressing effects of stimulant treatment and related changes in meal timing, as well as from physical activity, socioeconomic status, and parental or family dietary patterns, all of which may influence both carbohydrate exposure and ADHD-related outcomes. This issue has been repeatedly highlighted in previous reviews of ADHD and nutrition and remains one of the central reasons why causal interpretation is limited [10,11,73,88].

Methodological diversity also helps explain the inconsistency observed across exposure domains. Differences in exposure definitions, dietary assessment methods, ADHD outcome measures, study designs, and confounder adjustment limited direct comparability across studies and supported the use of a structured narrative synthesis rather than quantitative pooling. These issues support a cautious interpretation of the evidence and are addressed more fully in the limitations section.

A key strength of the present review is that it moves beyond broad “healthy versus unhealthy diet” framing and focuses specifically on carbohydrate-related exposures. This is valuable because prior ADHD nutrition literature has often grouped sugar, refined grains, soft drinks, and ultra-processed foods into wider Western-pattern constructs, making it difficult to determine whether carbohydrate-related characteristics are independently relevant [10,14,83,84,85,88]. By separating total carbohydrate intake from added sugars, GI/GL, and higher-quality carbohydrate indicators such as fiber and whole-grain patterns, the present review provides a more exposure-specific synthesis. In that sense, the review extends prior evidence by showing that the most consistent adverse signal is linked not to carbohydrate intake in general, but to poorer carbohydrate quality.

### 4.1. Limitations

Several considerations should be taken into account when interpreting the findings of this review. First, the included evidence base was methodologically heterogeneous and was dominated by observational, often cross-sectional, studies, which limits causal inference and leaves substantial scope for reverse causation [34,35,47,51,52,65]. Second, carbohydrate-related exposures were variably defined across studies, ranging from total carbohydrate intake and added sugars to sugar-sweetened beverages, glycemic index/load, fiber, and broader dietary patterns, which reduced comparability across studies and complicated exposure-specific interpretation [34,36,40,48,50,59,62]. Third, many studies relied on self- or parent-reported dietary intake and differed in the extent to which they distinguished total sugars from added sugars or controlled for key confounders such as socioeconomic status, parental or family dietary patterns, sleep, ADHD medication use, body mass index, physical activity, and overall diet quality [34,35,38,40,43,47,48,50,51,52,56,65]. Fourth, ADHD outcomes were not uniformly defined, with some studies using formal diagnostic criteria and others relying on symptom-based screening instruments [14,35,36,37,43,47,48,50,51,62,64]. Fifth, most intervention studies modified multiple aspects of diet simultaneously, limiting attribution of observed effects specifically to carbohydrate quantity or carbohydrate quality [71,73,74,76,77,79]. Sixth, restricting eligibility to English-language publications from 2015 to 2025 may have introduced language and publication-period bias, although this time frame was selected to reflect contemporary dietary patterns, food environments, and diagnostic practices. Seventh, inter-reviewer agreement was not quantified using Cohen’s kappa, which may limit the reproducibility assessment of the screening process. These considerations reduce certainty, but they do not negate the observed pattern that poorer carbohydrate quality was more consistently associated with ADHD-related outcomes than total carbohydrate intake.

### 4.2. Clinical Implications

The clinical implication should remain cautious and evidence-based. The available evidence does not justify framing carbohydrates overall as a primary causal factor in ADHD or using dietary modification as a stand-alone treatment. However, it supports the view that higher consumption of added sugars, sugar-sweetened beverages, and poorer-quality carbohydrate-rich dietary patterns is more frequently associated with greater ADHD symptom burden [14,34,35,36,38,43,48]. In practice, dietary guidance may reasonably focus on limiting sugar-sweetened beverages, sweets, and highly refined high-GI carbohydrate foods, while encouraging fiber-rich, minimally processed carbohydrate sources such as whole grains, legumes, fruits, and vegetables. Current ADHD clinical guidelines provide broad advice on balanced nutrition and lifestyle support, but limited carbohydrate-specific recommendations, highlighting a gap between emerging exposure-specific evidence and clinical practice [89,90]. Such recommendations should be framed as adjunctive nutritional support within multimodal ADHD care, ideally delivered with family/carer involvement and, where appropriate, dietitian input, rather than as a replacement for evidence-based pharmacological or behavioral treatment [11,34,35,36,43,48,49,89,90].

### 4.3. Future Prospects

Future research should address four main priorities. First, exposure definitions should be standardized, with clearer distinction between total carbohydrates, added/free sugars, refined carbohydrates, fiber, whole grains, and dietary GI/GL. Second, study design should be strengthened through well-powered prospective cohorts and randomized controlled trials with repeated dietary assessment, longer follow-up, transparent adherence reporting, and adequate adjustment for confounders such as medication use, sleep, physical activity, socioeconomic status, total energy intake, and overall diet quality. Third, mechanistic studies are needed to clarify whether carbohydrate quality influences ADHD-related outcomes through glycemic variability, insulin signaling, reward-related eating behavior, sleep–appetite regulation, inflammation, or gut–brain pathways. Fourth, future work should identify responsive subgroups, including individuals with emotional eating, reward sensitivity, sleep disturbance, metabolic vulnerability, or different ADHD presentations, in order to determine whether carbohydrate-quality modification may be more relevant for specific clinical profiles.

## 5. Conclusions

This systematic review indicates that carbohydrate quality, particularly higher intake of added sugars, sugar-sweetened beverages, and poorer-quality carbohydrate-rich dietary patterns, is more consistently associated with ADHD-related outcomes than total carbohydrate intake. What is currently supported by the evidence is a pattern of association: poorer carbohydrate quality is more frequently linked to greater ADHD symptom burden, while total carbohydrate intake alone shows inconsistent findings. What remains uncertain is whether these associations reflect independent effects of carbohydrate quality, broader dietary patterns, residual confounding, or bidirectional relationships between ADHD symptoms and food choices.

Evidence regarding glycemic index/glycemic load and fiber-related exposures remains suggestive but limited, and interventional studies are too heterogeneous to establish a clear carbohydrate-specific therapeutic effect. These findings support the consideration of reducing added sugars and improving carbohydrate quality as part of broader, adjunctive dietary strategies in ADHD management, but not as a primary or stand-alone treatment. Larger prospective studies and well-controlled dietary trials are needed to clarify causality, mechanisms, and responsive subgroups.

## Figures and Tables

**Table 1:** Characteristics of observational studies included in the systematic review.

No.	Included Study	Country	Study Design	Population	Carbohydrate-Related Exposure(s)	ADHD- Related Outcome	Direction of Association
1.	Yu et al., 2016 [34]	Taiwan	Observational	Children	Sugar-sweetened beverages	ADHD	Higher SSB intake associated with higher odds of ADHD
2.	Ríos-Hernández et al., 2017 [35]	Spain	Observational	Children	Sugar, candy, cola beverages, non-cola soft drinks; Mediterranean-diet adherence	ADHD diagnosis	Higher sugar/soft drink intake and lower Mediterranean-diet adherence associated with ADHD
3.	Yan et al., 2018 [36]	China	Observational	Preschool children	Processed, snack, and vegetarian dietary patterns	ADHD symptoms	Processed/snack patterns positively associated; vegetarian pattern inversely associated
4.	Abbasi et al., 2019 [37]	Iran	Observational	Children/adolescents	Western dietary pattern rich in processed meat, snacks, fat, salt	ADHD	Western pattern positively associated with ADHD
5.	Del-Ponte et al., 2019 [14]	Brazil	Birth cohort	Children	Sucrose consumption	ADHD incidence	No clear association between sucrose intake and incident ADHD
6.	Mian et al., 2019 [38]	Netherlands	Population-based cohort	Children	Overall diet quality	ADHD symptoms	ADHD symptoms predicted lower later diet quality, but not vice versa
7.	Borge et al., 2021 [39]	Norway	Pregnancy cohort	Mother–child cohort	Maternal and child diet quality	Child ADHD symptoms	Better maternal diet quality associated with slightly lower child ADHD symptom scores
8.	Alick et al., 2021 [40]	USA (NEST cohort)	Cohort	Mother–child cohort	Periconceptional high glycemic loading	Offspring attention-/ADHD-related behavior	Higher maternal glycemic loading associated with less favorable offspring behavior
9.	Lertxundi et al., 2022 [41]	Spain/Greece cohorts	Prospective cohort	Mother–child cohort	Maternal dietary inflammatory index	ADHD symptoms at age 4	More proinflammatory maternal diet associated with higher ADHD symptoms
10.	Kvalvik et al., 2022 [42]	Norway	Cohort	Mother–child cohort	Maternal sweetened carbonated beverage intake in pregnancy	Offspring ADHD symptoms	Weak positive association with offspring ADHD symptoms
11.	Lee et al., 2022 [43]	South Korea	Prospective cohort	Children aged 4 followed to 6 years	Sweet dietary pattern	ADHD symptoms	Sweet dietary pattern associated with higher attention-deficit/hyperactivity symptoms
12.	Rojo-Marticella et al., 2022 [44]	Spain	Observational	Children with and without ADHD	Food consumption and dietary patterns	ADHD and eating pattern differences	Dietary-pattern differences observed between ADHD and non-ADHD groups
13.	Akin et al., 2022 [45]	Turkey	Case–control	Children	Processed meat products, milk desserts, chocolate/sweets, snacks	ADHD	Children with ADHD consumed more processed meat, desserts, and chocolate/sweets
14.	Yan et al., 2023 [46]	China	Case–control	Children	Processed food-sweets patterns	ADHD	Processed food-sweets scores positively associated with ADHD risk
15.	Zhang et al., 2023 [47]	China	Cross-sectional	Schoolchildren	Sugar-sweetened beverage consumption	Hyperactivity	Positive dose–response association with hyperactivity risk
16.	Yingchankul et al., 2023 [48]	Thailand	Observational	Medical students	Added sugar from beverages	ADHD symptoms	Higher added sugar from beverages associated with higher ADHD symptoms
17.	Kim et al., 2024 [49]	Republic of Korea	Cohort	Early childhood cohort	Sweetened beverage consumption before age 2	Later ADHD	Early SSB exposure associated with increased later ADHD risk
18.	Solberg et al., 2024 [50]	Norway	Pregnancy cohort	Mother–child cohort	Prenatal maternal fiber intake	Offspring ADHD symptoms	Lower prenatal fiber intake associated with higher offspring ADHD symptom levels
19.	Kim et al., 2018 [51]	South Korea	Observational	Elementary school children	Soft drinks, fast food, instant noodles; unhealthy dietary habits	ADHD symptoms/K-ARS	Positive
20.	Chou et al., 2018 [52]	Taiwan	Case–control	Children with and without ADHD	Dietary/nutrient profile including refined grains and broader diet composition	ADHD diagnosis/status	Mixed/group differences
21.	Wang et al., 2019 [53]	Taiwan	Case–control	Children with and without ADHD	High-sugar/high-fat foods vs. healthier foods	ADHD diagnosis/status	Positive for nutrient-poor, sugary foods
22.	San Mauro Martín et al., 2018 [54]	Spain	Cross-sectional/observational	Children	Mediterranean diet adherence; cereal, pasta/rice, baked goods	ADHD diagnosis	Inverse for healthier pattern; adverse for poorer-quality foods
23.	Jamshidnia et al., 2021 [55]	Iran	Case–control	Children	Refined grains and food-group intake	ADHD/hyperactivity	Positive for less favorable food pattern
24.	Salvat et al., 2022 [56]	Iran	Case–control	Children with and without ADHD	Simple sugars, ready-made meals, overall dietary patterns	ADHD diagnosis/status	Positive for poorer-quality dietary pattern
25.	Ryu et al., 2022 [57]	South Korea	Longitudinal observational	School-age children	Dietary intake patterns including sugar/refined-carbohydrate-related intake	ADHD scores	Positive
26.	Wolff et al., 2019 [58]	Germany	Observational	Children and adolescents with ADHD	Candy and fruit-gum consumption	Hyperactivity/ADHD-related behavior	Positive
27.	Cendra-Duarte et al., 2025 [59]	Spain	Pregnancy cohort	Mother–child cohort	Maternal dietary glycemic index/glycemic load	Offspring attention-/ADHD-related behavior	Positive for higher GL/GI
28.	Wang et al., 2020 [60]	Taiwan	Case–control	Children with and without ADHD	Dietary patterns with carbohydrate-quality relevance	ADHD case–control status	Mixed/pattern differences
29.	Leventakou et al., 2022 [61]	Greece	Prospective cohort	School-age children	Eating behavior patterns	ADHD symptoms	Mixed/behavioral-pattern associations
30.	Darabi et al., 2022 [62]	Iran	Case–control	Children	Mediterranean diet adherence	ADHD odds	Inverse
31.	Darand et al., 2022 [63]	Iran	Case–control	Children	Plant-based dietary score	ADHD odds	Inverse/healthier score associated with lower odds
32.	Bayranj et al., 2025 [64]	Iran	Case–control	Children	MIND diet adherence	ADHD odds	Inverse
33.	San Mauro Martín et al., 2021 [65]	Spain	Observational	Children	Diet/lifestyle pattern including carbohydrate-relevant foods	ADHD	Mixed/poorer pattern adverse
34.	Darzi et al., 2022 [66]	Iran	Case–control	Children	Polyphenol-rich dietary intake as marker of dietary quality	ADHD odds	Inverse
35.	Navab et al., 2025 [67]	Iran	Case–control	Children	Energy-adjusted dietary inflammatory index	ADHD risk	Positive for more pro-inflammatory pattern
36.	Ehlers et al., 2019 [68]	Germany	Case–control/observational	Children and adolescents	Preference for sweets, candy, chewing gum	ADHD group differences	Positive/higher preference in ADHD group
37.	Abbasi et al., 2022 [69]	Iran	Case–control	Children	Diet cost as proxy for diet quality and food pattern	ADHD odds	Mixed/indirect dietary-quality link
38.	Leventakou et al., 2016 [70]	Greece	Cohort/observational	Early childhood cohort	Eating behavior	ADHD symptoms	Mixed

**Table 2:** Characteristics of interventional studies included in the systematic review.

No.	Included Study	Country	Study Design	Population	Carbohydrate- Related Intervention(s)	ADHD-Related Outcome	Intervention Effect
1.	Ghanizadeh and Haddad, 2015 [71]	Iran	Randomized controlled clinical trial	Children with ADHD	Overall dietary education/favorite vs. unfavorite diet modification	Inattention and hyperactivity/ impulsivity scores	Mixed; “un-favorite” diet showed no clear effect, but broader dietary guidance was investigated as an ADHD intervention
2.	Alonso Canal et al., 2019 [72]	Spain	Pilot intervention study	6 pediatric ADHD patients	Gluten-free diet for 4 months	CPT-II and reported ADHD symptom changes	Occasional ADHD-symptom improvement reported; no statistically significant CPT-II change
3.	Pelsser et al., 2020 [73]	Netherlands	Practice-based interventional study	Children with ADHD in specialized care	Few-foods diet (FFD) with reintroduction phase	ADHD and ODD symptom response	Positive; 34/57 (60%) were ADHD responders after the FFD
4.	Dölp et al., 2020 [74]	Germany	Open-label dietary intervention	Children/ adolescents with ADHD	Oligoantigenic diet (4 weeks) with reintroduction phase	ADHD Rating Scale IV	Positive; blinded/non-blinded ratings both supported improvement, with 5/8 responders among complete data
5.	Hontelez et al., 2021 [75]	Netherlands/Germany-linked research setting	Open-label intervention trial	Children with ADHD	Few-foods diet	ADHD symptom change with neurofunctional correlates	Positive symptom change following FFD; study focused on relation between symptom improvement and brain-function change
6.	Khoshbakht et al., 2021 [76]	Iran	Randomized controlled clinical trial	Children aged 6–12 years with ADHD	DASH-style diet	ADHD symptom severity	Positive; authors concluded a DASH-style diet might improve ADHD symptoms
7.	Lawrence et al., 2022 [77]	New Zealand/Australia-linked ADHD nutrition research context	Non-randomized feasibility study	Children with ADHD	Microbiome-targeted dietary intervention	Feasibility, acceptability, and ADHD-related clinical monitoring	Preliminary/mixed; intervention was feasible and well tolerated, but this was primarily a feasibility study
8.	Walz et al., 2022 [78]	Germany	Long-term follow-up intervention study	Children previously completing a 4-week oligoantigenic diet	Oligoantigenic diet plus individualized long-term nutrition	ADHD Rating Scale IV at follow-up	Positive; long-term symptom improvement persisted compared with pre-diet baseline
9.	Huberts-Bosch et al., 2025 [79]	Netherlands	Randomized controlled trial	165 children aged 5–12 years with ADHD	Elimination diet vs. healthy diet; non-randomized care-as-usual comparator arm	Combined parent/teacher ADHD and emotion-regulation response after 5 weeks	Mixed; both dietary approaches showed responses, but elimination diet was not superior to healthy diet
10.	Eder et al., 2025 [80]	Germany	Follow-up intervention study	Children and adolescents with ADHD	Oligoantigenic diet	Core ADHD symptoms, especially impulsivity	Positive; authors concluded the diet may have long-term therapeutic potential, especially for impulsivity

## Data Availability

No new data were created or analyzed in this study. Data sharing is not applicable to this article.

## Abbreviations

The following abbreviations are used in this manuscript:

ADHD	Attention-deficit/hyperactivity disorder
DSM	Diagnostic and Statistical Manual of Mental Disorders
FFD	Few-foods diet
GI	Glycemic Index
GL	Glycemic Load
ICD	International Classification of Diseases
NOS	Newcastle–Ottawa Scale
PECOS	Population–Exposure–Comparator–Outcome–Study design
PRISMA	Preferred Reporting Items for Systematic Reviews and Meta-Analyses
PROSPERO	International Prospective Register of Systematic Reviews
RoB 2	Cochrane Risk of Bias 2
ROBINS-I	Risk Of Bias In Non-randomised Studies—of Interventions
